# Cultural Humility: A Proposed Model for a Continuing Professional Development Program

**DOI:** 10.3390/pharmacy8040214

**Published:** 2020-11-13

**Authors:** Jennifer L. Cox, Maree Donna Simpson

**Affiliations:** 1 Three Rivers Department of Rural Health, Charles Sturt University, Orange, NSW 2800, Australia; 2 School of Biomedical Sciences, Charles Sturt University, Orange, NSW 2800, Australia; masimpson@csu.edu.au

**Keywords:** cultural humility, cultural safety, continuing professional development

## Abstract

Continuing professional development (CPD) is an essential component of professional practice for registered health practitioners to maintain and enhance knowledge, skills and abilities. There are many topics that practitioners may pursue relevant to their practice environment, and, in recent years, providing culturally safe and respectful practice is an emerging area of need. Unfortunately, many health professionals, whilst willing to offer cultural safe healthcare, may be uncertain of how to enact that practice. The World Health Organisation recognises attainment of the highest possible standard of health as a basic human right, and cultural safety is increasingly becoming an expectation of health professionals. To address this need and the insufficiency of support in the literature, the authors have presented a discussion paper on various aspects of cultural safety and the underlying constructs, such as cultures, that support it. The discussion takes into account core constructs that signpost the path to cultural safety and recognises the role and accountability of all levels of the healthcare system, not merely the practitioner. Finally, we propose a model program for a cultural humility CPD activity incorporating pre-work, online modules, interactive workshop, reflection on professional practice and a post-workshop evaluation.

## 1. Introduction

In recent years, there has been increasing recognition of cultural safety as a framework for addressing inequality in healthcare and improving health outcomes of indigenous people culminating in embedding of cultural safety in healthcare standards [1,2]. As many nations become more culturally diverse, there are often challenges to providing culturally safe care. There may be additional challenges as nations welcome refugees and immigrants into their communities.

Australia, which lies in the southern hemisphere, is an island nation with 25,499,884 people [3]. It is also recognised as the most multicultural or ethnically nation in the world [4]. This is evidenced by data which establish that 26% of people permanently resident in Australia are born overseas and 49% have at least one parent born overseas. This is different from other countries with, for example, the proportion of overseas-born people being 14% in the United States, 22% in Canada, and 23% in New Zealand [5]. In addition, it has been identified that people in Australia can choose to worship freely from 100 religions that are practised. Further, 300 ethnic/cultural groups are identified in Australian culture. Indigenous Australians, our nation’s First peoples, constitute 3.3% of the nation’s population [6].

Subsequent to World War II, waves of migrants and refugees have made Australia their new home. There are people from European nations such as Greece and Italy, later people from Vietnam, India and more recently, the Middle East. Thus, health professionals in Australia face many challenges in providing care. Increasingly, health professionals experience the need to develop not only cultural competence, but inter-cultural competence strategies to provide culturally safe care. According to Laverty and colleagues, “culturally safe care results where there is no inadvertent disempowering of the recipient, indeed where recipients are involved in the decision making and become part of a team effort to maximise the effectiveness of the care” [2].

**Figure 1:**
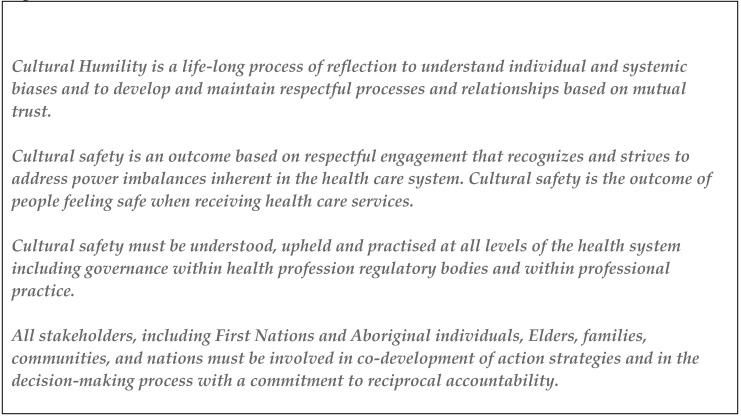
Core constructs in the development of culturally safe practice.

In 2017, the Registrar of the College of Pharmacists of British Colombia (BC) signed the “Declaration of Cultural Safety and Humility in Health Services Delivery for First Nations and Aboriginal Peoples in BC” as a means of improving BC pharmacy professionals’ work with First Nations and Aboriginal people [7]. This declaration was based on the following guiding principles (Figure 1):

We identify that the path to culturally safe practice is a journey not a destination, and navigating the path may not be straightforward. The aim of this paper is to unpack the key constructs of the guiding principles, namely cultural safety and humility, on which this declaration was based and highlight the crucial role of CPD.

## 2. Cultural Competence

Although the World Health Organisation (WHO) recognises attainment of the highest possible standard of health as a basic human right, there can be many challenges along the path to achieving this goal [8]. Globally, there are longstanding racial/ethnic and urban/rural disparities in health and healthcare, which are well recognised and are well documented [9,10,11]. The impact on individual’s health as a result of these sociocultural factors has been increasingly recognised globally.

The concept of cultural competence was introduced in the 1980s and identified as a means towards health equity for minority or disadvantaged groups [1]. A variety of definitions for cultural competence can be found in the literature, but broadly, the concept is generally been framed in an individual context which “focuses on the capacity of the health worker to improve health status by integrating culture into the clinical context” [12]. More recently, Jowsey [13] proposed that there are in fact three different types (zones) of cultural competence, namely: surface competency—the shallowest level of competence where the focus is on what people say and do, and demonstrating culturally specific knowledge; bias twilight zone—a deeper level where the focus is on increasing self-awareness of implicit bias; the confronting midnight zone—where there is recognition of the impact of power and privilege on one’s worldview.

Of concern, many models of cultural competence entail little or no acknowledgement of the fluidity or subjectivity of culture, nor of the power differential in any relationship between a healthcare provider and patient/client [14]. Much of the contemporary cultural competence training is developed on the basis of differences between “our” culture and “theirs”, a focus on mastery of “other” cultures/prevailing cultural belief systems [14]. This can result in a “recipe book” approach to cultural interactions, an approach which is potentially based on stereotypes or generalisations and thus may be neither applicable nor appropriate to many people. Whilst it may be easy to consider one has “achieved” cultural competence, it must be remembered that cultures are dynamic and “that every culture is heterogeneous, either temporally or spatially” [15]. It could therefore be argued that it is impossible for any single healthcare professional to be “competent” in all cultures, since not only do people vary by cultural background but also by gender, by age, by life stage, by concurrent morbidities/chronic diseases, by birth status or as a refugee or migrant in a foreign nation. Each of these factors can impact the power imbalances inherent in the practitioner–patient relationship. Curtis and colleagues argued that health equity simply cannot be achieved without acknowledging the notion of “power” within cultural competency frameworks. Failure to do so “…perpetuate[s] deficit discourses that place responsibility for problems with the affected individuals or communities…” [1]. The role and power of healthcare professionals and healthcare organisations are an important consideration as each, effectively, are gate keepers of access for the disadvantaged.

## 3. Cultural Safety

Culturally safe and responsive care is now recognised as integral to reducing, and ultimately eliminating, health disparities, and the expectation for health practitioners to provide culturally safe and responsive care is clearly articulated in professional practice standards. For example, the Pharmaceutical Society of Australia (PSA) Professional Practice Standards state that “pharmacy practice must be underpinned by professionalism and ethical behaviour, and should reflect principles of equity, patient-centred care, cultural safety, evidence-based practice and the quality use of medicines (QUM)” [16]. With regards to the cultural safety and health equity aspect of these Standards, the following criteria are listed:

- Recognising and responding to the specific health needs of Aboriginal and Torres Strait Islander people, and other populations identified as experiencing healthcare inequity.
- Ensuring that all individuals are treated with respect and consideration of their beliefs, cultures and practices.
- Delivering healthcare equitably.
- Conducting regular review of self, co-workers and the workplace for cultural and social responsiveness.

Cultural safety moves beyond cultural awareness and acknowledgement of cultural differences. Rather, cultural safety focuses on the experience of the patient/healthcare recipient and seeks to address the power imbalances and inequitable social relationships that exist in healthcare. Importantly, cultural safety also places emphasis on the role of healthcare organisations in creating and maintaining culturally safe environments [17]:

[In Australia] Cultural safety is determined by Aboriginal and Torres Strait Islander individuals, families and communities. Culturally safe practise is the ongoing critical reflection of practitioner knowledge, skills, attitudes, practising behaviours and power differentials in delivering safe, accessible and responsive healthcare free of racism [18].

By acknowledging and responding to power differentials and dynamics, practitioners move away from ethnocentrism and, ultimately, are able to shift between worldviews and cultural frames of Reference [19].

In summary, cultural safety recognises the role and accountability of all levels of the healthcare system. Culturally safe healthcare is “defined by patients and their communities and should be measured through progress towards achieving health equity” [1]. However, as noted in the Commitment Statement from College of Pharmacists of British Colombia, cultural safety cannot be achieved without enacting cultural humility.

## 4. Cultural Humility

Cultural humility describes the life-long, active and reflective process (rather than being about achieving an end-goal) that recognises power imbalances between health professionals and patients:

Cultural humility incorporates a lifelong commitment to self-evaluation and critique, to redressing the power imbalances in the physician-patient dynamic, and to developing mutually beneficial and non-paternalistic partnerships with communities on behalf of individuals and defined populations [20].

A move from cultural competence to cultural humility, of necessity, entails a move away from health professional mastery towards individual accountability and acknowledgement (and mitigation) of power imbalances between practitioner and patient. In practice, a practitioner seeking to embody cultural humility enacts flexibility [21] and a commitment to active engagement in life-long self-reflection and self-critique. In doing so, “they are flexible and humble enough to assess anew the cultural dimensions of the experiences of each patient” [20], thereby encouraging the patient to also be their teacher. As the practitioner learns about their patient, they are both a student and teacher in this relationship. According to Chang, et al. [22], the exploration, understanding and appreciation of a patients’ socio-cultural context, values and beliefs in regard to health, in turn, results in improved patient satisfaction and health outcomes.

Culturally responsive communication is an important and expected component of this process. As stated in the PSA Competency National Competency Standards (Domain 2, Standard 2.1) [23], a pharmacist must “Respect the personal characteristics, rights, preferences, values, beliefs, needs and cultural and linguistic diversity of patients and other clients, including Aboriginal and Torres Strait Islander peoples.”

Effective culturally responsive communication requires clinicians to acknowledge that culture influences a patient/clients their view of and reaction to health messages [24]. For example, it is recognised that some “folk” illnesses such as empacho (gastrointestinal obstruction) in Hispanic cultures, have a “culturally constructed description of the pathophysiology” [25] resulting in wide variation of intra- and inter-cultural beliefs regarding the cause of the blockage, symptoms and the required treatment. Failure to acknowledge the influence of culture on beliefs about health and illness [25] can result in misinterpretation of symptoms, inappropriate treatment or misunderstanding of treatment goals [26]. The same considerations apply even when the practitioner may be a member of the same cultural group as the patient, since cultural groups have some commonalities but vary by age, gender and so forth. An example of this can be seen where a member of one aboriginal “tribe” goes to work in a region where patients come from a different “nation”.

Poor health literacy can also impact a patient’s ability to appraise health information, make health decisions [27] and understand the care and/or medications that are being prescribed [28]. Linguistic differences and low health literacy (of patients) can negatively impact development of trust due to lack of shared vision [29,30]. Therefore, effective communication addresses both culture and health literacy. As identified in the systematic review by Minnican and O’Toole [26], the specific communication skills and behaviours required to achieve effective cross-cultural communication include the ability to listen, clarifying understanding, inclusion and/or acknowledgement of family, limiting the use of jargon, and using inclusive language. Recommendations for health-literate sensitive communication include using plain language, breaking down instructions into small specific steps, using pictures or diagrams and ensuring that any printed information or resources are written at or below fifth-grade reading level. These precautions should be applied universally, that is, to all patients regardless of their literacy levels [27,31].

In summary, simply understanding the concept of cultural humility is not enough to ensure translation into culturally safe practice. Given the ongoing, active nature of cultural humility, we submit that there is a need for explicit continuing professional development education (CPD) for health practitioners in this area.

## 5. Cultural Humility CPD

The ongoing nature of the cultural humility process aligns well with the “lifelong learning” principle/purpose of CPD [32]; however, frameworks for the development and evaluation of cultural humility as CPD models, particularly in the pharmacy discipline, are scarce. Whilst CPD models for cultural safety training in health disciplines such as midwifery [33] are well documented, cultural humility training is an often-overlooked component in health professional development.

In their 2017 paper, van Hoof and Meehan [34] argued that simply increasing clinician knowledge by learning new information or skills, although necessary, is insufficient to generate sustained practitioner behavioural change and improved patient outcomes. Rather, a new paradigm for CPD where educational or quality improvement programs focussed on practice change was needed. Under this new paradigm, CPD programs would have predisposing, enabling and reinforcing elements as the central premise of their design. Predisposing components would communicate information about an opportunity for improvement. The enabling component would develop competence or facilitate the desired change in practice (relating to the opportunity for change), and the reinforcing component would assist in recall of competence or reinvigorate change in practice.

Interactive workshops are identified as one such intervention that can be effective in accomplishing the “enabling” and “reinforcing” components of such CPD programs and can result in moderately large changes in professional practice [35]. Inclusion of at least 25% interactive elements in CPD learning interventions has been found to shift the learning from a passive to an active process [36]. In the case of cultural humility CPD, we propose that adopting a culturally responsive framework [37], underpinned by cultural humility as a reimagined model of practice, is crucial.

**Figure 2:**
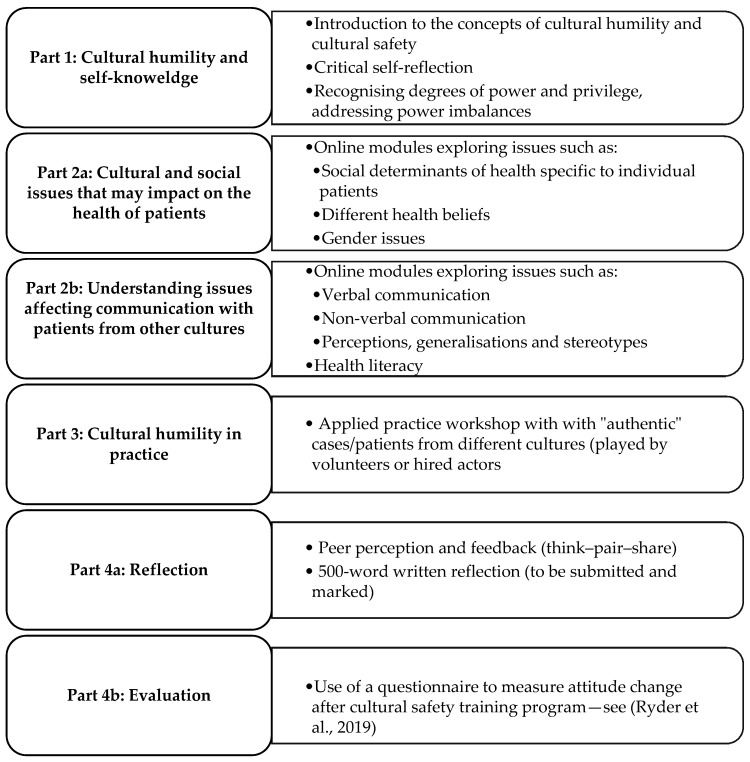
Outline of proposed cultural humility continuing professional development (CPD) program.

Figure 2 outlines the proposed interactive program which features a combination of face-to-face and on-line elements.

Parts 1 and 2 of this program focus on “turning inward” [38] whereby participants explore their own cultural and professional identities, consider privilege and power structures and acknowledge how their background and experiences of privilege help, or hinder, their connection to patients. Prior to the workshop, participants would be asked to reflect on their own biases and assumptions about race, gender and sexual orientation by completing an online tool such as Harvard’s Project Implicit (https://implicit.harvard.edu/implicit/). Individual and organisational questions for assessing cultural humility [14] are then used within the program for further reflection of the value we attribute to input from patients and what we learn about our practice and ourselves by engaging with patients who are different to us. Participants will be given an opportunity to share examples of challenging cultural encounters as well as success stories to help gain a deeper understanding of the ongoing, reflective nature of cultural humility.

Part 2 of the program is a series of case-based studies, developed collaboratively with authentic patients, to help develop practitioners’ understanding of issues that may affect the health of and communication with patients from different cultures and within the same culture.

Part 3 is an applied practice workshop that gives participants an opportunity to reinforce their learning by intentionally facilitating effective encounters with “authentic” patients. Despite best intentions, health practitioners may “lack insight into how their behaviours affect patients or how to modify those behaviours” [39]. In authentic practice or in real life, situations, challenges and successes are contextualised and “made sense of” by reflection—contemplating the outcomes and looking forward.

Finally, Part 4 focuses on reflection and evaluation. According to the stages of change model (Table 1), people move through a series of stages when modifying behaviour. The five stages of this model differ according to an individual’s intention and behaviour [40].

Reflective journaling and small-group discussions are embedded throughout the course to stimulate critical reflection [20]. Small breaks (5–10 min) will be included in each stage of the workshop to enable participants to write reflections, notes and ideas [42]. In the final stage of the program (Part 4), participants will be given a chance to discuss their conclusions with a view to writing a 500-word reflection piece to be submitted for marking within four weeks of completing the program.

A key element of the proposed program would be the development of a personal learning plan (PLP) and evidence portfolio to document the training program and behaviour changes in practice, provide evidence of evaluation of outcomes and capture visible evidence of achievement of reflection and capacity to share/disseminate. As such, this cultural humility program aligns with the requirements of Group 3 CPD activities as described by the Pharmacy Board of Australia:

Group 3: quality or practice—improvement facilitated (three Board CPD credits per hour of activity)

Descriptor: activities where an assessment of existing practice (as an individual or within a pharmacy practice), and the needs for, and barriers to changes in this practice, is carried out before the development of a particular activity. As a result, the activity addresses identified continuing professional development needs with a reflection post-activity to evaluate practice change or outcomes resulting from the activity. Such an activity most likely will extend over a number of weeks or months [43].

To contextualise each workshop as far as possible and allow it to be adaptable to different learners and professions, participants would be asked to complete a pre-workshop survey eliciting their reasons for attending and their goals. Additional resources can be then offered throughout the program to meet learner expectations.

## 6. Conclusions

The need for every health professional to acknowledge and “step outside” their own health beliefs in order to ascertain and assess culturally relevant information from or about the patient is widely acknowledged. Cultural humility is an active and ongoing process that focusses on self-reflection and facilitates the client becoming the teacher and co-creating their health experience and health outcomes. It encourages interactions that are respectful of cultural beliefs, and, thus, we believe that it is a necessary component of all culturally safe healthcare provision. It therefore needs to be included in the ongoing CPD of health practitioners. Culturally safe healthcare, however, also requires input of community members. Thus, any educational intervention would require collaboration and participation from patients and community leaders from all cultural groups. An interactive program such as the one discussed in this paper could enhance the provision of culturally safe care and meet CPD requirements for practising and intern pharmacists.

## Figures and Tables

**Table 1:** Stages of change model.

Stage	Stage Characteristics
Precontemplation	Not thinking about change; satisfied with status-quo.
Contemplation	Thinking about change, but not yet sure.
Preparation	Would like to change; may be planning and trying out changes
Action	Beginning to change. Specific, overt behavioural modifications have been made. Observable changes take place.
Maintenance	Have successfully implemented change. Consolidation of the behaviours initiated during the action stage.

Source: Adapted from [41].
